# When STEMI Isn’t STEMI: Cardiac Arrest from Aortic Valve Papillary Fibroelastoma – A Case Report

**DOI:** 10.5811/cpcem.50489

**Published:** 2026-01-16

**Authors:** Mohammad Wasim Mohammad Zouhir Kanaa, Shahd H I Abbastanira, Esraa Mohamed Abdullah Elameen, Sarah Emad Alsultan

**Affiliations:** Dubai Health, Department of Emergency Medicine, Dubai, United Arab Emirates

**Keywords:** papillary fibroelastoma, cardiac arrest, acute coronary syndrome mimic, transesophageal echocardiograph, case report

## Abstract

**Introduction:**

Cardiac arrest remains a major global cause of mortality, with both structural and non-structural cardiac abnormalities implicated. While ischemic heart disease is a common etiology, rare conditions such as papillary fibroelastoma can also result in life-threatening events through embolization or coronary obstruction. Timely recognition and advanced cardiac imaging, particularly transesophageal echocardiography, are essential in such atypical presentations.

**Case Report:**

A 61-year-old female with a history of ischemic heart disease presented to the emergency department following an out-of-hospital cardiac arrest. Initial electrocardiogram (ECG) demonstrated anterior ST-elevation myocardial infarction, which resolved on repeat ECG, prompting reconsideration of the underlying cause. The patient achieved return of spontaneous circulation twice and eventually self-extubated. Further investigation, including cardiac imaging, revealed a mobile mass on the aortic valve intermittently obstructing the left main coronary artery. The mass was surgically resected and histologically confirmed as a papillary fibroelastoma. The patient recovered fully with no neurological or cardiac complications.

**Conclusion:**

This case highlights the importance of maintaining a broad differential diagnosis in patients presenting with cardiac arrest, especially when ischemic changes are transient or unexplained. Although benign, papillary fibroelastoma can lead to sudden death due to embolization or coronary obstruction. Emergency physicians should be aware of such rare but treatable causes and consider early use of advanced cardiac imaging when standard presentations do not align with the clinical picture.

## INTRODUCTION

Cardiac arrest remains a leading cause of mortality worldwide, with cardiac etiologies accounting for the majority of cases. While structural heart diseases such as cardiomyopathies, congenital anomalies, and heart failure are well-recognized contributors, non-structural conditions like Brugada syndrome and long QT syndrome also increase the risk of fatal arrhythmias.^1^ Rarely, external compression of coronary arteries by masses or malformations can lead to fatal events, especially when classic risk factors are absent.

Papillary fibroelastoma (PFE) is the most common primary tumor of cardiac valves, comprising approximately 75% of valvular tumors. Although histologically benign, its mobile and friable nature poses significant risk of embolization and dynamic obstruction, potentially resulting in stroke, myocardial infarction, or sudden cardiac death.^2^ Early detection is crucial, and transesophageal echocardiography remains the diagnostic modality of choice due to its superior sensitivity.^3^

We present the case of a 61-year-old woman with a history of ischemic heart disease and systemic lupus erythematosus who experienced out-of-hospital cardiac arrest. Her initial electrocardiogram (ECG) suggested anterior ST-elevation myocardial infarction (STEMI), which resolved on repeat testing. The patient achieved return of spontaneous circulation twice and ultimately self-extubated. Subsequent imaging identified a mobile aortic valve mass, later confirmed as papillary fibroelastoma.

## CASE REPORT

A 61-year-old female with a history of ischemic heart disease, hypertension, and systemic lupus erythematosus was brought to the emergency department (ED) following an out-of hopsital cardiac arrest with ongoing cardiopulmonary resuscitation (CPR). According to her daughter, she collapsed suddenly at approximately 9 pm with agonal breathing. Bystander CPR was initiated immediately, and paramedics arrived about 10 minutes later, providing Advanced Cardiac Life Support (ACLS). During prehospital resuscitation, she underwent 14 cycles of CPR, achieved return of spontaneous circulation twice, and experienced two episodes of unstable ventricular tachycardia requiring defibrillation. The estimated total downtime before sustained return of spontaneous circulation was approximately 38 minutes.

*CPC-EM Capsule*

What do we already know about this clinical entity?

*Papillary fibroelastomas (PFE) are rare cardiac tumors that can cause embolization or obstruct coronary ostia, leading to myocardial ischemia or arrhythmias.*

What makes this presentation of disease reportable?

*Intermittent obstruction of the left main coronary ostium by a mobile PFE causing recurrent cardiac arrest and transient ST-Elevation Myocardial Infarction (STEMI) is extremely rare.*

What is the major learning point?

*Transient STEMI or unexplained cardiac arrest should prompt evaluation for structural causes like PFE when common etiologies do not fully explain the presentation.*

How might this improve emergency medicine practice?

*Emergency medicine clinicians should consider structural lesions in unexplained arrest or transient electrocardiogram changes and use early advanced cardiac imaging to avoid missed diagnoses.*

**Image 1:**
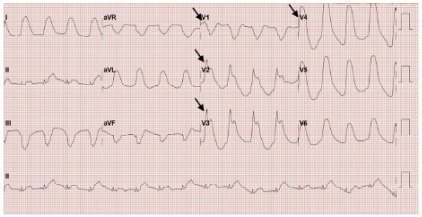
Initial electrocardiogram demonstrating ST-segment elevation in leads V1–V4 (arrows), consistent with anterior ST-segment elevation myocardial infarction.

**Image 2:**
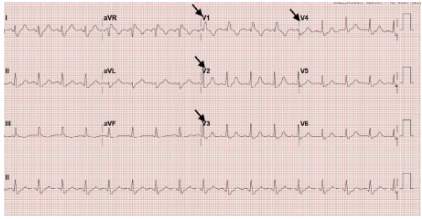
Repeat electrocardiogram post return of spontaneous circulation shows resolution of the ST-segment elevations and a new right bundle branch block (arrows).

Upon arrival to the ED, the patient remained in cardiac arrest with ongoing CPR and a laryngeal mask airway in place. Return of spontaneous circulation was achieved in the ED, after which she was intubated for airway protection and connected to a mechanical ventilator. Induction was performed with etomidate and succinylcholine, and sedation was maintained with midazolam and fentanyl. The initial ECG, obtained post-return of spontaneous circulation, demonstrated anterolateral STEMI (Image 1), prompting cardiology consultation and activation of the STEMI code. A repeat ECG five minutes later showed resolution of the ST elevations and a new right bundle branch block (Image 2). Shortly thereafter, the patient experienced another cardiac arrest with a non-shockable rhythm; return of spontaneous circulation was regained after two cycles of CPR. She subsequently developed hypotension, prompting discontinuation of midazolam and fentanyl and initiation of a norepinephrine infusion. Peak troponin reached 390 nanograms per liter (ng/L) (reference range: < 34 ng/L), indicating significant myocardial injury.

After achieving return of spontaneous circulation, the patient regained consciousness and self-extubated, demonstrating spontaneous breathing and intact neurological function. A non-contrast brain computed tomography (CT) was unremarkable, and point-of-care echocardiography demonstrated normal left ventricular systolic function without regional wall motion abnormalities. Due to transient ECG changes and hemodynamic instability, pulmonary embolism was considered and ruled out via a negative CT pulmonary angiogram (CTPA). Aortic dissection was also considered but deemed unlikely due to the transient ST-segment elevation on ECG and the lack of radiological evidence of dissection on CTPA.

**Image 3:**
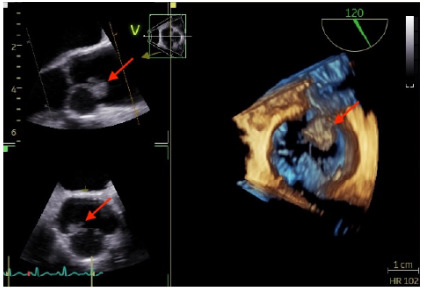
Transesophageal echocardiography showing a 1.5 × 1.0 centimeters, highly mobile lesion attached to the left coronary cusp of the aortic valve (arrows). The mass has a very small neck and intermittently obstructed the left main coronary ostium.

The patient was admitted under cardiology for further management and evaluation with percutaneous coronary intervention. Coronary angiography revealed non-flow-limiting stenoses in the mid left anterior descending (LAD) and distal right coronary arteries (RCA). A departmental echocardiogram identified an aortic root mass, further characterized by transesophageal echocardiography as a 1.5 × 1.0-cm lesion attached to the left coronary cusp of the aortic valve (Image 3), later identified as PFE. The mass was surgically resected, and the patient made a full recovery without post-arrest neurological deficits.

## DISCUSSION

Sudden cardiac arrest refers to the abrupt cessation of cardiac activity, triggered by malignant ventricular arrhythmias such as sustained pulseless ventricular tachycardia and ventricular fibrillation,^4^ resulting in hemodynamic collapse. Coronary artery disease, the underlying cause in over 75% of sudden cardiac death cases in developed countries,^5^ often leads to these arrhythmias. While the causes of sudden cardiac death vary by age, acute coronary syndrome accounts for nearly 50% of cases in individuals ≥ 40 years of age.^6^

This case highlights a rare and diagnostically challenging cause of sudden cardiac arrest. The initial suspected cause of the arrest was STEMI, based on the patient’s presentation and past medical history. This prompted immediate resuscitation as per the ACLS protocol and ACS management of acute coronary syndrome. However, after the second return of spontaneous circulation, a repeat ECG showed resolution of ST elevations, prompting reconsideration of the diagnosis and broadening the differential. Hypoxia was excluded, as the patient was intubated and mechanically ventilated. She experienced fluctuating blood pressure and several episodes of cardiac arrest. As sedation was tapered, she regained consciousness and self-extubated. These events, with the evolving ECG findings and fluctuating hemodynamic status, prompted consideration of other potential causes, including pulmonary embolism, which was ruled out via a negative CTPA, and aortic dissection, which was also excluded given the transient ST-segment elevation on ECG and the absence of dissection on CTPA. After stabilization, angiography showed non-flow-limiting LAD and RCA stenosis. Transesophageal echocardiography revealed a 1.5 × 1.0-cm mobile mass on the left coronary cusp intermittently obstructing the left main ostium, confirmed through histopathology as a PFE and was resected surgically.

Recurrent ventricular tachycardia and cardiac arrest were likely due to intermittent obstruction of the left main coronary ostium by the mobile PFE. This mechanism is supported by previous reports where transient ischemia from fibroelastoma-induced obstruction triggered arrhythmias.^7^ Return of spontaneous circulation between arrests likely indicates intermittent resolution of the obstruction, permitting transient restoration of coronary perfusion.

Similar cases of papillary fibroelastoma causing coronary obstruction have been reported in the literature. Talari et al described a 73-year-old woman with recurrent cardiac arrests and an inferior STEMI caused by a PFE obstructing the anterior coronary cusp, ultimately requiring surgical resection.^8^ Raheela et al reported a 68-year-old woman who developed acute myocardial infarction due to a PFE on the right coronary cusp, with distal coronary embolization resulting in ST-segment elevation.^9^ Ismaiel et al described a 39-year-old male presenting with inferior STEMI and a right coronary cusp PFE detected by echocardiography and surgically removed.^10^ Ramirez et al reported a 63-year-old woman presenting with angina; myocardial perfusion imaging showed anterior ischemia, and cardiac CT angiography revealed a PFE on the left coronary cusp causing intermittent left main coronary artery occlusion during systole and early diastole, which resolved after surgical resection.^11^ Most previously reported cases involve the right coronary artery or its branches, whereas obstruction at the left main ostium, as seen in our patient and in Ramirez et al, is less commonly reported. These cases highlight the importance of considering structural cardiac lesions, such as PFE, in patients presenting with transient ischemic ECG changes or unexplained cardiac arrest.

Although primary cardiac tumors are rare (0.02–0.45%), their potential to cause fatal events through embolization or coronary obstruction warrants heightened clinical awareness. Papillary fibroelastoma is the most common valvular tumor and second only to myxoma overall.^12^ Despite being benign and slow-growing, PFEs are clinically significant due to their high mobility and embolic risk.^13^ Rarely, they may intermittently obstruct coronary ostia, triggering arrhythmias and cardiac arrest,^7^ as demonstrated in this case. Transesophageal echocardiography remains the diagnostic gold standard, offering near-100% sensitivity, especially for small tumors.^14^ Surgical resection is recommended for mobile or symptomatic PFEs to prevent recurrence and sudden death.^15^.

This case underscores the importance of maintaining a broad differential diagnosis in cases of sudden cardiac arrest and incorporating advanced cardiac imaging early in the evaluation of unexplained cases of cardiac arrest, especially when there is rapid evolution in clinical status, ECG findings, and hemodynamic parameters. Rare structural causes such as PFE should be considered when common etiologies are ruled out or clinical findings are inconsistent with typical presentations.

## CONCLUSION

This case highlights the importance of maintaining a high index of suspicion for rare structural causes of cardiac arrest when common diagnoses like acute coronary syndrome do not fully explain the presentation. Although papillary fibroelastoma is histologically benign, it can cause sudden death through coronary obstruction. Early use of advanced cardiac imaging, particularly transesophageal echocardiography, is essential for timely diagnosis. Emergency physicians should remain especially vigilant when there is rapid evolution in clinical status or ECG changes to identify and manage these rare but potentially treatable causes of cardiac arrest.
